# Supplementary material for: Synergism between IL-33 and MRGPRX2/FcεRI Is Primarily Due to the Complementation of Signaling Modules, and Only Modestly Supplemented by Prolonged Activation of Selected Kinases
Source: Cells. 2023 Nov 24;12(23):2700. doi: 10.3390/cells12232700 (PMC10705352; doi:10.3390/cells12232700)
Supplement: Supplementary file 1 [file cells-12-02700-s001.zip › cells-2637374-supplementary.pdf]

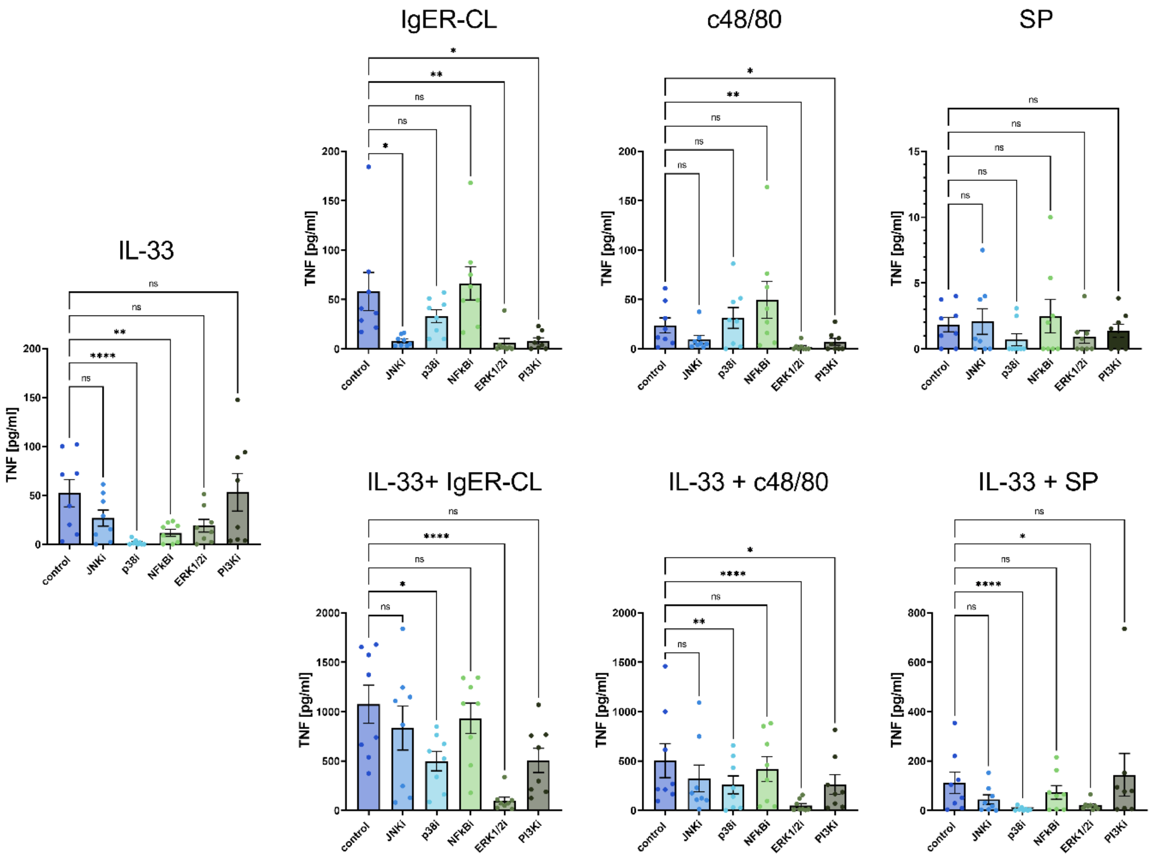

Figure S1 Contribution of distinct modules to TNF stimulation in dependence of the stimulus. The same date as presented in the main manuscript in Figure 1 but in another set-up is shown to allow for a direct statistical comparison between the different inhibitors upon individual types of stimulation.
Skin MCs were stimulated by IgER-CL (crosslinking, AER-37 at 0.2 μg/ml) or SP (30 μM) or c48/80 (10 μg/ml) or IL-33 (20 ng/ml) for 24 h, TNF was quantified in the resulting supernatants by ELISA. Control; vehicle was used instead of inhibitor. cells were pretreated with the respective inhibitors for 15 min and then stimulated. ERK1/2 inhibitor SCH772984 at 10 μM, JNK inhibitor SP600125 at 10 μM, p38 inhibitor SB203580 at 10 μM. NF-κB inhibitor BAY11-7082 at 1 μM, PI3K inhibitor Pictisilib at 10 μM. * $p < 0.05$, ** $p < 0.01$, **** $p < 0.0001$. Friedman test with Dunn's multiple comparison test. i = inhibitor

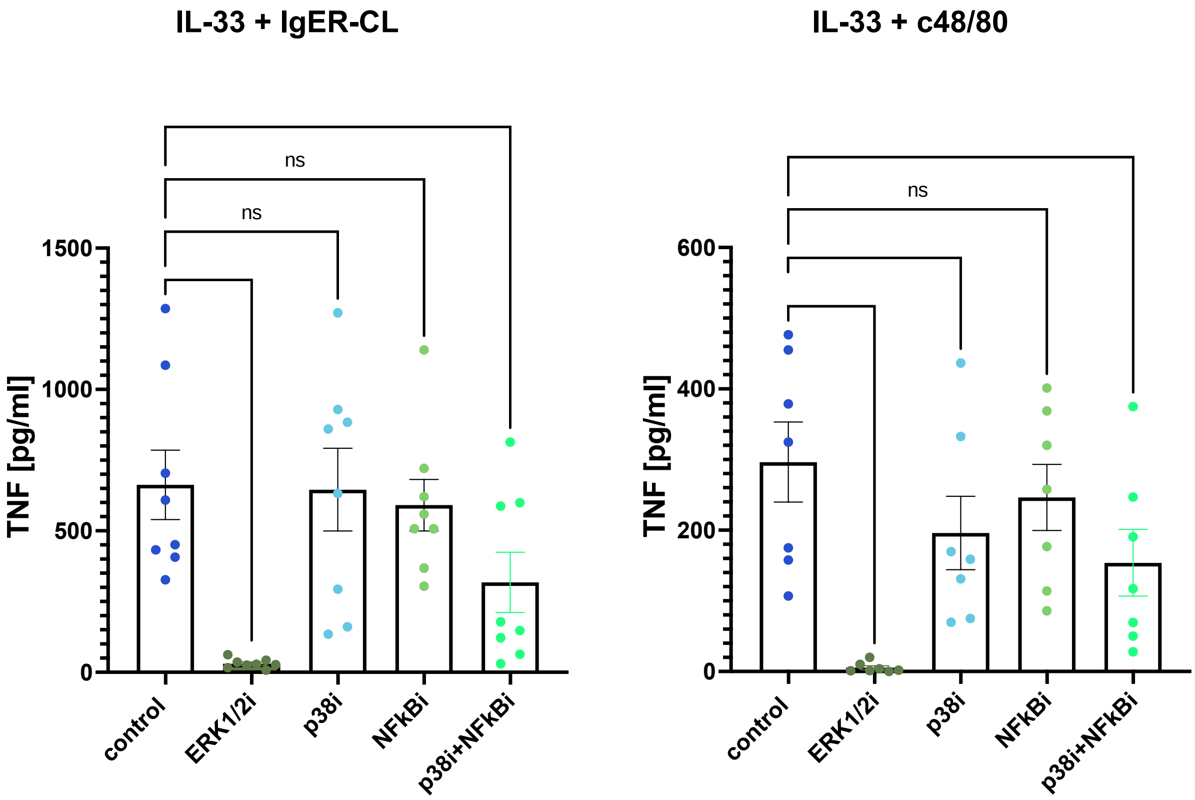

Figure S2 Simultaneous inhibition of NF-κB and p38 does increase the impact of each inhibitor alone but does not reach the inhibitory potency of ERKi.
Skin MCs were stimulated with a combination of IL-33 (20 ng/ml) and IgER-CL (crosslinking, AER-37 at 0.2 μg/ml) or IL-33 (20 ng/ml) and c48/80 (10 μg/ml) for 24 h, TNF was quantified in the resulting supernatants by ELISA. Control; vehicle was used instead of inhibitors. cells were pretreated with the respective inhibitors for 15 min and then stimulated. ERK1/2 inhibitor SCH772984 at 10 μM, p38 inhibitor SB203580 at 10 μM. NF-κB inhibitor BAY11-7082 at 1 μM. The same concentrations were used in the combined setting. * $p < 0.05$, ** $p < 0.01$, *** $p < 0.001$, **** $p < 0.0001$. RM one-way ANOVA with Holm-Šídák's multiple comparisons test.

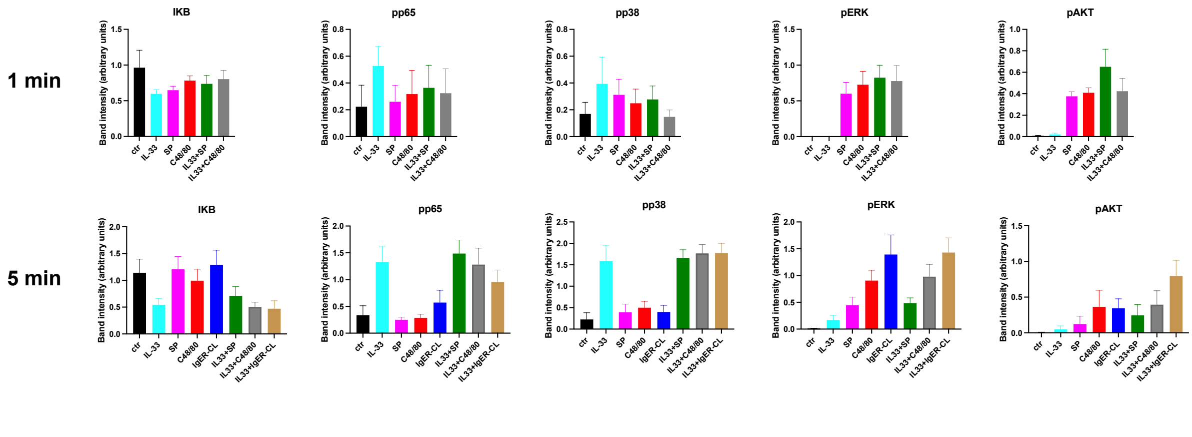

Figure S3. IL-33, FcεRI and MRGPRX2 triggered signaling events are barely affected by each other at early times. Skin MCs were stimulated by IL-33 (20 ng/ml), IgER-CL (crosslinking, AER-37 at 0.2 μg/ml) or SP (30 μM), c48/80 (10 μg/ml) or the specified combinations for 1 min (top) or 5 min (bottom). The degradation or phosphorylation of signaling components were detected by immunoblot. Cyclophilin B (CyclB) and α-actinin served as the loading controls. Mean ± SEM of n = 2-4.
